# Using Geospatial Analyses of Linked Electronic Health Records and Tobacco Outlet Data to Address the Social Determinants of Smoking

**DOI:** 10.5888/pcd16.190186

**Published:** 2019-11-14

**Authors:** Scott D. Siegel, Madeline M. Brooks, Bayo M. Gbadebo, James T. Laughery

**Affiliations:** 1 Value Institute, Christiana Care Health System, Newark, Delaware; 2 Helen F. Graham Cancer Center and Research Institute, Christiana Care Health System, Newark, Delaware

**Figure:**
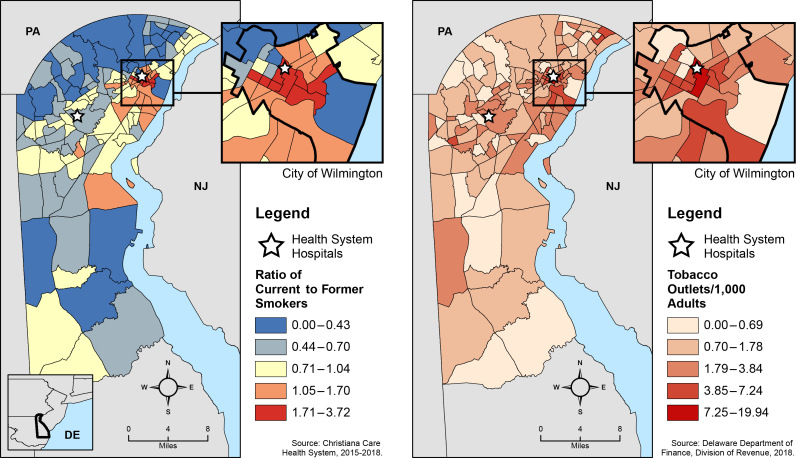
These maps depict the ratio of current to former smokers within a hospital-based population and tobacco outlet density (tobacco outlets/1000 adults) by census tract in New Castle County, Delaware. The countywide ratio of current to former smokers is 0.73. In Wilmington, the county’s largest and most populous city, the ratio of current to former smokers is 1.33. Wilmington also experiences a tobacco outlet density more than double that of the county (3.51 vs 1.62).

## Background

The medical community now widely recognizes that social determinants of health (SDOH) have a significant effect on population health (1). If health systems are to move beyond a focus on traditional clinical services to successfully addressing SDOH, new models of care delivery are needed to guide this transition. Toward that end, the Centers for Disease Control and Prevention (CDC) developed a conceptual framework to facilitate collaboration between health systems and public health practitioners. This framework prioritizes increasing the adoption of clinical care that has a strong public health evidence base, innovating new forms of care delivery outside of clinical settings, and implementing community-wide interventions (2). As a first step toward operationalizing this framework, health systems can look beyond the “walls of the hospital” to develop an understanding of SDOH within their communities. By linking patients’ electronic health record (EHR) clinical data to area-level measures (3), geospatial analyses can be employed to inform the development of novel interventions that address local SDOH.

To illustrate the potential of this approach, we linked patient-level smoking status and address data from a Mid-Atlantic health system EHR to local area–level SDOH measures. Despite notable successes in tobacco control over the last 50 years or more, smoking remains the leading preventable cause of morbidity and mortality in the US, with slower declines in smoking rates for people of low socioeconomic status (4). Hospital-based smoking cessation interventions are effective (5); however, people of low socioeconomic status are more likely to reside in neighborhoods with more tobacco outlets and other challenges (6), which can undermine smoking cessation efforts (7). Therefore, we included area-level measures of socioeconomic status and tobacco outlet exposure as indicators of the social determinants of smoking status.

## Data Sources and Map Logistics

We included all adult patients with a smoking history admitted to Christiana Care Health System (CCHS) in New Castle County, Delaware, from January 1, 2015, through June 30, 2018, who resided in New Castle County. Smoking status (current, former, never) was assessed through a standardized interview administered at admission by the patient’s inpatient nurse and documented in the EHR. Patient addresses were cleaned and geocoded using ArcGIS 10.6 (Esri) (match rate = 94%). To adjust for geographic variation in the number of patients with a smoking history who were hospitalized at CCHS, a ratio of current to former smokers was calculated for each census tract. Tobacco outlet addresses were obtained from a public state business license database (8) and geocoded to calculate tobacco outlet density (TOD) for each census tract (number of tobacco outlets/1,000 adults). Patient-level tobacco outlet exposure was calculated as each patient’s Euclidean distance (in miles) to the nearest tobacco outlet and the number of tobacco outlets within a half-mile radius of their address. American Community Survey data provided census tract poverty and race/ethnicity characteristics (9). Census tracts were classified as high poverty if the percentage of residents who lived below the poverty line was equal to or greater than the 75th percentile and predominant minority if a nonwhite racial or ethnic group constituted the highest proportion of the tract population. Choropleth maps were created for smoker ratios and TOD by using natural breaks classification. Independent Samples *t* tests and χ^2^ tests were used to compare TOD by census tract classification and current versus former smokers on demographic, neighborhood, and tobacco outlet exposure characteristics.

## Highlights

These maps depict a strong association between smoking status and tobacco outlet exposure, particularly when contrasting Wilmington to the county at large. Of the 22,112 patients included in this analysis, 9,303 (42%) were current and 12,809 (58%) were former smokers, yielding a current-to-former-smoker ratio of 0.73 across the county’s 130 census tracts. The smoker ratio across Wilmington’s 25 census tracts was 1.33, 82% higher than the county. That is, for every 100 former smokers, there were 133 current smokers in Wilmington and 73 in the county overall. Similarly, Wilmington’s TOD was more than double the county overall (3.51 vs 1.62). Notably, CCHS was located in the census tract with the greatest TOD in the county and in proximity to 42 tobacco outlets within a half-mile radius. More generally, TOD was significantly higher in census tracts classified as high poverty (3.37 vs 1.42, *P* < .001), predominant minority (3.21 vs 1.56, *P* < .001), and both high poverty and predominant minority (3.45 vs 1.61, *P* < .001). Compared with former smokers, current smokers were significantly younger and more likely to be male and racial/ethnic minorities and to reside in census tracts characterized as high poverty, predominant minority, or both (Table). Furthermore, compared with former smokers, current smokers lived, on average, 0.10 miles closer to the nearest tobacco outlet, approximately the equivalent of 2 Wilmington city blocks, and in proximity to >70% more tobacco outlets within a 1/2-mile radius of their address.

**Table:** Characteristics of Hospitalized Current and Former Smokers (N = 22,112) in New Castle County, Delaware^a^

Variable	Current Smokers	Former Smokers	Total
**Total, n (%)**	**9,303 (42.1)**	**12,809 (57.9)**	**22,112**
**Demographics **			
**Age, mean (SD)**	50.9 (15.7)^c^	68.6 (15.6)	61.1 (17.9)
**Male, n (%)**	5,079 (54.6)^d^	6,718 (52.4)	11,797 (53.4)
**Race, n (%)**			
White	6,074 (65.3)^c^	9,676 (75.5)	15,750 (71.2)
Black	2,837 (30.5)^c^	2,718 (21.2)	5,555 (25.1)
Other	392 (4.2)^c^	415 (3.2)	807 (3.7)
**Ethnicity, n (%)**			
Hispanic/Latino	438 (4.7)^c^	415 (3.2)	853 (3.9)
Non-Hispanic/Latino	8,865 (95.3)^c^	12,394 (96.8)	21,259 (96.1)
**Census Tract^b^**			
Living in high-poverty census tract, n (%)	2,918 (31.4)^c^	2,485 (19.4)	5,403 (24.4)
Living in predominant minority census tract, n (%)	2,763 (29.7)^c^	2,144 (16.7)	4,907 (22.2)
Living in high-poverty, predominant minority census tract, n (%)	2,155 (23.2)^c^	1,518 (11.9)	3,673 (16.6)
**Tobacco Exposure**			
Miles to nearest tobacco outlet, mean (SD)	0.4 (0.5)^c^	0.5 (0.5)	0.4 (0.5)
Tobacco outlets within ½ mile of home, mean (SD)	9.8 (13.9)^c^	5.7 (9.7)	7.4 (11.8)

Abbreviations: SD, standard deviation.

a Demographic data and home address were extracted from the electronic health records for 22,112 adult patients with a self-reported history of smoking who were admitted to the Christiana Care Health System from January 2, 2015, through June 30, 2018, and resided in New Castle County, Delaware. *P* values were calculated by using χ^2^ tests for categorical variables and independent samples *t* tests for continuous variables.

b Census tract characteristics were obtained from 2016 American Community Survey 5-year averages. Census tracts were designated as high-poverty if they were ≥75th percentile for percentage of residents living below the poverty line and designated as predominant minority if a nonwhite racial or ethnic group constituted the highest proportion of the tract population. Patients were geocoded by home address and assigned the characteristics of the census tract in which they resided.

c Significant at *P* < .001.

d Significant at *P* = .002.

These findings extend prior community-based survey research on tobacco outlet exposure (6) to a health system population by linking area-level measures to patient-level EHR smoking history data. Greater tobacco outlet exposure can undermine smoking cessation efforts by increasing exposure to point-of-sale marketing and other smoking cues, easing access to cigarettes, and contributing to pro-smoking attitudes (7). Hospitalization represents an opportunity for smokers to make a quit attempt in a smoke-free environment with ready access to treatment. Unfortunately, returning to a neighborhood with high tobacco outlet exposure upon discharge can increase the likelihood of relapse.

The primary limitation of this snapshot is that it cannot support causal inferences. Without quit dates and contemporaneous addresses, it is unclear whether former smokers from this population were more likely to quit when living in neighborhoods with lower tobacco outlet exposure and higher socioeconomic status. Temporal considerations aside, greater TOD may contribute to higher rates of smoking, more demand for cigarettes may drive greater TOD, or “third variables” such as disinvestment may contribute to both the greater use of cigarettes to cope with living in low-socioeconomic status areas and more lenient zoning standards regarding tobacco outlets.

## Action

This snapshot can stimulate novel smoking cessation initiatives aligned with the CDC framework. At the clinical level, the results underscore the importance of ensuring access to evidence-based smoking cessation interventions given the SDOH many smokers face. In addition, behavioral interventions specifically designed to reduce reactivity to smoking cues (10) may prove uniquely beneficial for smokers with greater tobacco outlet exposure. Future research can evaluate whether such adjuvant treatments improve quit rates for these patients. At the community level, the fact that one of the CCHS hospitals was located in the census tract with the greatest TOD in the county would support extending smoking cessation programming to quite literally just outside the walls of the hospital (eg, partnering with local community organizations, deploying community health workers). At the population level, further research is needed to evaluate whether regulating TOD reduces smoking rates in low-socioeconomic status neighborhoods (7). Partnering with the local health department and advocacy organizations may facilitate such evaluation efforts. Taken together, this snapshot portrays how linking EHR and area-level data can guide more effective collaborations between health systems and public health practitioners to address the SDOH.
